# Innovative computational tools provide new insights into the polyploid wheat genome

**DOI:** 10.1007/s42994-023-00131-7

**Published:** 2024-02-07

**Authors:** Yongming Chen, Wenxi Wang, Zhengzhao Yang, Huiru Peng, Zhongfu Ni, Qixin Sun, Weilong Guo

**Affiliations:** Frontiers Science Center for Molecular Design Breeding, Key Laboratory of Crop Heterosis and Utilization, Beijing Key Laboratory of Crop Genetic Improvement, China Agricultural University, Beijing, 100193 China

**Keywords:** Polyploid wheat, Genome complexity, Functional genomics, Tool development

## Abstract

Bread wheat (*Triticum aestivum*) is an important crop and serves as a significant source of protein and calories for humans, worldwide. Nevertheless, its large and allopolyploid genome poses constraints on genetic improvement. The complex reticulate evolutionary history and the intricacy of genomic resources make the deciphering of the functional genome considerably more challenging. Recently, we have developed a comprehensive list of versatile computational tools with the integration of statistical models for dissecting the polyploid wheat genome. Here, we summarize the methodological innovations and applications of these tools and databases. A series of step-by-step examples illustrates how these tools can be utilized for dissecting wheat germplasm resources and unveiling functional genes associated with important agronomic traits. Furthermore, we outline future perspectives on new advanced tools and databases, taking into consideration the unique features of bread wheat, to accelerate genomic-assisted wheat breeding.

## Introduction

Bread wheat (*Triticum aestivum*; genome BBAADD 2*n* = 6*x* = 42), also known as common wheat, originated from two distinct rounds of polyploidization. It is one of the most widely cultivated cereal crops worldwide and provides about a fifth of the calories consumed by humans (Shiferaw et al. 2013). Nevertheless, both climate change and the limited availability of arable land have raised concerns about sustainable crop production (Atlin et al. 2017; Springmann et al. 2018). Developing elite crop cultivars to maximize grain yield and quality is an important way to resolve the global food issue and feed the growing population (Borrill et al. 2019; Springmann et al. 2018). Obtaining a comprehensive understanding of the functional genome underlying agronomic traits can assist genome design and genetic improvement, leading to improved agricultural production (Atlin et al. 2017; Hickey et al. 2019; Wang et al. 2018).

Research in wheat functional genomics progressed slowly in the past until the release of the first reference genome (IWGSC 2018), which greatly advanced molecular genetics and functional genomics and facilitated the development of enhanced cultivars with superior agronomic traits (Adamski et al. 2020). Meanwhile, the reduction in sequencing costs and the advancement in sequencing technologies have already led to a significant increase in wheat multi-omics resources such as genome, transcriptome, and epigenome (Concia et al. 2020; Ramirez-Gonzalez et al. 2018; Walkowiak et al. 2020). Such a substantial amount of biological data enables wheat researchers to gain a deep understanding of gene functions and regulatory mechanisms associated with important agronomic traits such as disease resistance, adaptability, and grain yield (Xiao et al. 2022; Xu et al. 2022). Nevertheless, handling such vast data, especially in the context of bread wheat, poses numerous challenges for researchers. Efficient tools and advanced methodologies are needed to decipher the diverse types of data, thereby enabling the transformation of the raw data into functional genomic knowledge for wheat. Here, we provide an overview of the progress and development of our recently developed tools and databases that consider the complexity and unique characteristics of the wheat genome. We also illustrate how these computational tools and databases could provide new insights into the polyploid wheat genome and enhance further genomics-assisted genetic improvement, and discuss future perspectives for designing genomic tools.

## The complexity and unique features of the bread wheat genome

**Fig. 1:**
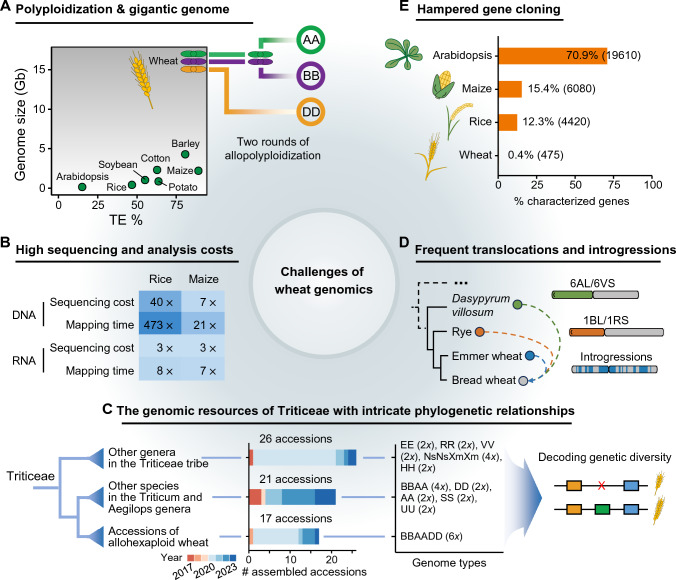
The challenges of wheat functional genomics research. **A** Bread wheat originated from two successive rounds of polyploidization and has a much larger and more complex genome than other economically important crops. The abscissa represents the proportion of transposable elements, and the ordinate represents genome size. **B** High computation and sequencing costs for genomic analysis due to a gigantic wheat genome. The numbers indicate the ratio of the mapping time or cost of wheat to rice or maize. We used 5 × WGS data to calculate the time cost for analyzing WGS data. 6 GB of RNA-seq data was used in rice and maize, while 18 Gb of that was used in wheat. Sixteen threads were used for each analysis. **C** Increasing reference genome and pan-genome resources for bread wheat and its relatives provide great opportunities while posing challenges for efficient exploration of genomic variation for wheat improvement. The middle plots show the number of accessions with chromosome-level genome assemblies, their publication years, and their genome composition. **D** Frequent introgressions and translocations that occurred during wheat evolution have increased the complexity of the wheat genome. Tshe schematic diagram shows the known translocations 6AL/6VS and 1BL/1RS in wheat, as well as frequent introgressions from tetraploid wheat. **E** Statistics of currently characterized genes in wheat, rice, maize, and Arabidopsis. The numbers in parentheses represent the total number of cloned genes. The genes in wheat, rice, maize, and Arabidopsis were acquired from the wheatOmics, funRiceGenes, maizeGDB, and TAIR databases, respectively

Powerful genomic tools can assist in decoding the polyploid wheat genome. However, the majority of existing tools and databases were designed for species that generally have diploid and small genomes and overlooked the unique features of the wheat genome (Canto-Pastor et al. 2021). The distinct characteristics of the wheat genome present challenges in interpreting and computing and call for designing and developing novel computational tools (Fig. 1). The main challenges of wheat genomics are described below.

### Polyploidization and a gigantic genome

The allohexaploid bread wheat evolves through two rounds of polyploidization, whose genome is about 16 Gb in size and is one of the largest ones in plants (Fig. 1A). It evolved through two successive rounds of polyploidization. The first polyploidization event between *T. urartu* (genome AA 2*n* = 2*x* = 14) and a diploid species related to *Aegilops speltoides* ssp. *speltoides* (genome BB 2*n* = 2*x* = 14) produced tetraploid wild emmer wheat (*T. turgidum* ssp. *dicoccoides*; genome BBAA 2*n* = 4*x* = 28). The second polyploidization event between *Ae. tauschii* ssp. *tauschii* (genome DD 2*n* = 2*x* = 14) and domesticated tetraploid wheat produced bread wheat (Levy and Feldman 2022; Marcussen et al. 2014 ;Wang et al. 2023). The genome plasticity of bread wheat is a key factor in its success under domestication, and nowadays bread wheat comprises about 95% of the overall wheat harvest (Dubcovsky and Dvorak 2007). The large genome size, high similarity between subgenomes, and a high proportion of repetitive sequences (about 85% of the genome) pose challenges for genomic study (IWGSC 2014). Additionally, the effects of polyploidy on phenotypic variation, such as dominance, dosage effect, and functional redundancy, make the understanding of important agronomic traits in wheat particularly challenging and intriguing (Borrill et al. 2019).

### High sequencing and analysis costs

For allohexaploid wheat with a large genome, analyzing massive genomic data to identify genetic variations and understand gene functions requires high costs as well as robust and efficient computational tools and skilled data analysts. To illustrate the computational and analytical costs specific to wheat, we compared the high-throughput sequencing cost and data analysis time in wheat with those in rice and maize (Fig. 1B). The genome size of bread wheat is 40 times that of rice and 7 times that of maize, leading to a proportionally higher DNA sequencing cost. Furthermore, because wheat has three times the total number of coding genes compared to those of rice and maize, the RNA sequencing cost is nearly tripled. The alignment of sequencing data increased even more strikingly. We extracted sequencing data with the same depth for these three species from previously published resequencing and RNA-seq datasets (Chen et al. 2023b; Wang et al. 2022) and assessed the mapping costs. The mapping time of DNA sequencing data in wheat is 473 times that of rice and 21 times that of maize. For RNA-seq, the mapping time in wheat is 8 times that of rice and 7 times that of maize. Thus, the high costs of sequencing, computation, and storage for bread wheat are unneglectable, driving innovation in developing more efficient and scalable computing tools.

### The genomic resources of Triticeae with intricate phylogenetic relationships

Bread wheat, together with the important cereal crop relatives like barley (*Hordeum vulgare*) and rye (*Secale cereale*), are the members of the Triticeae tribe in the grass family (Poaceae) (Feldman and Levy 2015). The relatives of wheat, including crops and wild species, exhibit extensive genetic diversity and retain many favorable traits related to stress resistance and yield, which can serve as potential gene pools for further genetic improvement. In recent years, the number of available genome assemblies in the Triticeae tribe has been continuously increasing (Fig. 1C). To date, there have been available chromosome-level genome assemblies for 17 accessions of allohexaploid wheat, 21 accessions from other species in the Triticum and Aegilops genera, and 26 accessions from other genera in the Triticeae tribe (Chen et al. 2020; Xiao et al. 2022). The increasing assemblies in Triticeae make it possible to unveil evolutionary history and genomic variations of functional genes at the pan-genomic level, thereby enhancing our understanding of the improvement potential of wheat (Laugerotte et al. 2022; Li et al. 2021; Melonek and Small 2022; Zhou et al. 2021). Nevertheless, the vast amounts of assemblies available and the inconsistent assembly and annotation pipeline limit comparative genomic analysis. Additionally, these closely related species have different origins and varying ploidy levels. The analytical pipelines often struggle to keep up with the amount of raw data produced (Berger and Yu 2023). As the number of genome assemblies increases, there is generally an exponential, rather than linear, growth in computational requirements. Lack of effective tools hindered the exploration of the genomic resources of bread wheat accessions and relatives.

### Frequent introgressions and translocations

During both natural and artificial hybridization in wheat evolution history, the genome has undergone substantial introgressions and translocations, highlighting the reticulate nature of wheat evolution (Wang et al. 2022) (Fig. 1D). In bread wheat, more than 430 blocks longer than 1 Mb have been reported to have diversity in their wild emmer ancestor, five of which were even longer than 100 Mb and encompassed the centromeric regions (Cheng et al. 2019). Additionally, the identification of introgressions from wild relatives into bread wheat revealed a cumulative size of approximately 709 Mb and 1,577 Mb in landraces and cultivars, respectively. A large fraction of those introgression fragments also showed an association with important traits. For example, two large-scale introgressions, the 1BL/1RS and 6AL/6VS translocations, are utilized to improve traits such as resistance to biotic and abiotic stress and grain yield (Wang et al. 2017; Xing et al. 2021). Those frequent introgression and translocation events have greatly shaped the genetic diversity in wheat germplasm, calling for advanced tools beneficial for performing population genetic analyses and developing new varieties that fully utilize the plasticity of the wheat genome (Przewieslik-Allen et al. 2021; Yang et al. 2022b).

### Hampered gene cloning

The genetic understanding of many important agronomic traits that control phenotypic variations in bread wheat has progressed slowly (Borrill et al. 2019; Gao et al. 2019; Liang et al. 2021), despite that wheat genomic research has made tremendous strides in recent years and is closer to the level of research in rice and maize. Due to the polyploidy nature and genome feature of wheat, constructing high-resolution genetic maps and deciphering intricate genetic basis underlying traits become more difficult. Linkage disequilibrium within the genome-wide association study (GWAS) panel decays over relatively long distances, greatly hampering the ability of GWAS to detect quantitative trait loci (QTL) and identify candidate genes in wheat (Pang et al. 2020; Wang et al. 2023b). Many wheat genes are present as two or three functional homoeologs, resulting in the fact that the phenotypic consequences of variation at a single homoeolog can be masked by redundant copies on the other subgenomes, making the study of quantitative traits such as GWAS particularly challenging in polyploid wheat (Borrill et al. 2019). Additionally, reverse genetics approaches cannot identify functionally redundant genes and require a high cost to obtain homozygous artificial mutations (Wu et al. 2021). In the most well-studied model plant, *A. thaliana*, about 70.9% of the genes in its genome have been studied (Lamesch et al. 2012) (Fig. 1E). Additionally, in the model crops maize (Portwood et al. 2019) and rice (Yao et al. 2018), 15.4% and 12.3% of all genes have also been characterized, respectively. In contrast, only 0.4% of the genes in the wheat genome have been characterized (Ma et al. 2021), indicating that the function of most elements in the wheat genome has not been revealed. Limited functional gene information hinders the application of modern breeding approaches.

## The development of advanced tools and databases for wheat genomics

**Fig. 2:**
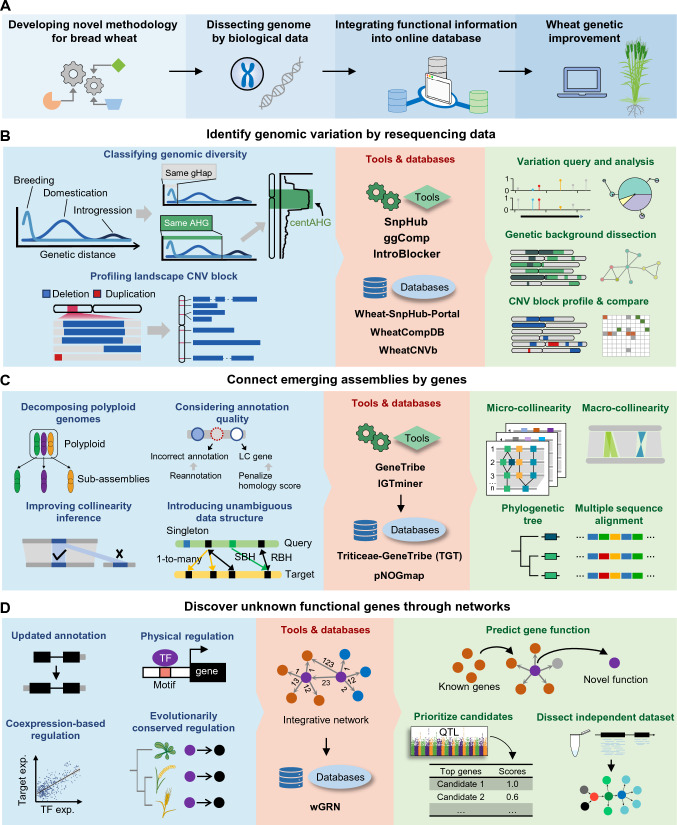
Genomic tools and methodologies targeting the wheat genome. **A** Considering the unique characteristics of the wheat genome, developing innovative computational tools can effectively assist in bread wheat improvement. **B** Two tools named ggComp and IntroBlocker, that consider the distribution of genetic distance among accessions, and a CNV block-based marker atlas can classify genomic variations into haplotypes that diverged in different evolution and breeding processes of wheat. centAHG represents the large, stable AHG block observed in the central zone of the chromosome. SnpHub, a webserver framework, was developed for light query and variation analysis. Several corresponding databases and websites named wheat-SnpHub-portal, WheatCompDB, and WheatCNVb were further designed to ease accessing the published wheat variation data, the dissection of haplotypes, and the identification and profiling of CNV blocks, respectively. **C** By addressing the unique characteristics of bread wheat and its relatives, such as polyploidy, collinearity, and poor gene annotation, GeneTribe and IGTminer were developed to perform homology and collinearity inference for the whole genome and the nuclear organellar genes, respectively. The corresponding databases, namely TGT and pNOGmap, were developed for the community to access data. The tools can effectively explore pan-genomic resources and aid in the evolutionary analysis of genes and chromosomes, such as collinearity, phylogeny, and multiple sequence alignment analysis. LC gene represents low-confidence gene. RBH represents reciprocal best hit. SBH represents single-side best hit. **D** By updating genome annotation, integrating evidence of gene expression networks and physical regulatory networks, and adding powerful evolutionary information, an integrative regulatory network database, wGRN, was developed for the community to effectively discover phenotypic trait-associated functional genes, dissect regulatory mechanisms, and prioritize candidate genes in GWAS. TF represents transcription factor

Recently, we have developed a series of genomic tools to unveil comprehensive functional information about gene function and its phenotypic variations and evolution by targeting the unique features and challenges of the polyploid wheat genome and dissecting data from multiple omics levels (Fig. 2A). The corresponding online platforms and databases were also developed for users to access these data conveniently and freely. Here, we categorized resources based on usage and illustrated their potential applications (Table 1).

**Table 1:** Summary of selected genomic databases and tools in wheat

Tool name	Type	Synopsis	Link	References
Wheat-SnpHub-Portal	Database	A genomic variation database for wheat and its progenitors	http://wheat.cau.edu.cn/Wheat_SnpHub_Portal/	(Wang et al. 2020)
Triticeae-GeneTribe (TGT)	Database	A homology and collinearity database for Triticeae	http://wheat.cau.edu.cn/TGT/	(Chen et al. 2020)
WheatOmics	Database	A platform combining multi-omics data to accelerate wheat functional genomics studies	http://wheatomics.sdau.edu.cn/	(Ma et al. 2021)
WheatCompDB	Database	A genetic relationship database for wheat germplasm	http://wheat.cau.edu.cn/WheatCompDB/	(Yang et al. 2022b)
wGRN	Database	A platform using wheat integrative regulatory networks to guide functional gene discovery	http://wheat.cau.edu.cn/wGRN/	(Chen et al. 2023b)
pNOGmap	Database	A database for nuclear organellar genes in Poaceae	http://wheat.cau.edu.cn/pNOGmap/	(Chen et al. 2023c)
wLNCdb	Database	A long non-coding RNA database for wheat	http://wheat.cau.edu.cn/wLNCdb/	(Zhang et al. 2023)
WheatCNVb	Database	A copy number variation block database for wheat germplasm	http://wheat.cau.edu.cn/WheatCNVb/	Unpublished
Wheat Grain Translatome Browser	Dataset	A browser for analyzing the translatomic data of developing wheat grains	http://wheat.cau.edu.cn/wheat_grain_translatome_browser/	(Guo et al. 2023)
SnpHub	Tool	A web server framework for exploring large-scale genomic variation data	http://guoweilong.github.io/SnpHub/	(Wang et al. 2020)
GeneTribe	Tool	A tool for performing collinearity-incorporating homology inference	https://chenym1.github.io/genetribe/	(Chen et al. 2020)
ggComp	Tool	A pair-wised comparison method to identify similar genetic regions and shared CNV regions	https://zack-young.github.io/ggComp/	(Yang et al. 2022b)
IntroBlocker	Tool	A semi-supervised algorithm for ancestral genomic block dissection	https://wangzihell.github.io/IntroBlocker/	(Wang et al. 2022)
IGTminer	Tool	A tool for identifying the evolutionary trajectories of nuclear organellar genes	https://chenym1.github.io/IGTminer/	(Chen et al. 2023c)

### Dissect genomic diversity hierarchically

Since the release of the first wheat genome (IWGSC 2018), more than a thousand wheat accessions have been genotyped using whole genome sequencing (WGS) (Cheng et al. 2019; Guo et al. 2020; Hao et al. 2020; Niu et al. 2023; Schulthess et al. 2022; Walkowiak et al. 2020; Wang et al. 2022; Yang et al. 2022b; Zhao et al. 2023; Zhou et al. 2020), which constitutes a rich data resource while raising challenges in interpreting those big data. To facilitate efficient exploration of this variation data, a web server framework, SnpHub, was developed for providing fast and lightweight variation query and population genetics analysis (Wang et al. 2020) (Fig. 2B), enabling users to easily determine population structure as well as allele frequency and distribution for any genomic region, free of programming knowledge. Additionally, a portal website, wheat-SnpHub-portal, was further designed to continuously collect and integrate published variation datasets in the Triticum and Aegilops genera with SnpHub (Wang et al. 2020).

The wheat genome accumulated extensive and intricate types of variations throughout its complex evolution. A phenomenon of stratified genetic distance distribution was observed among wheat varieties, and the Gaussian mixture distribution was used to model the mixture distribution of genetic distances into three levels, corresponding to the genetic divergence that cumulated through the diversification of wild emmer wheat lineages (~ 0.5 Mya), wheat domestication (~ 10 Kya), and modern wheat breeding (~ 100 ya), respectively, according to estimated divergence times. Two methods, ggComp (Yang et al. 2022b) and IntroBlocker (Wang et al. 2022), were proposed for classifying genetic resources according to diversity levels into corresponding modern germplasm resources and ancestral haplotypes, respectively. With IntroBlocker, accessions with a genetic distance lower than the threshold (~ 1 variation per 1 Kb) to distinguish different wild emmer wheat were grouped as ancestral haplotype groups (AHGs) in sliding windows along chromosomes, and a mosaic pan-ancestry haploblock map spanning both tetraploid and hexaploid wheat was constructed, enabling to explain the dispersed emergence and protracted domestication of polyploid wheat. Additionally, as a special characteristic of the Triticeae genome, centAHG was identified to represent the large stable AHG blocks across the central zone of a chromosome, ranging from 85 to 295 Mb, which is supposed to play a role in the chromosome backbone, which could be hard to break during the long evolution process, even including domestication and modern breeding. With ggComp, wheat accessions with genetic diversity lower than threshold (~ 1 variation per 100 Kb) to distinguish distinct germplasm resources, which consisted of random mutations cumulated for thousands of years before modern breeding, are grouped together, denoted as germplasm resource type-based haplotype (gHap). Furthermore, a genomic-based germplasm network (GGNet) integrating hundreds of wheat varieties was constructed, supporting interrogating pairwise gHap relationships among accessions by revealing the genetic relationships at multiple scales, tracing the breeding history of specific genome fragments, and inferring potential pedigrees. A user-friendly online database, WheatCompDB was designed for exploring gHaps in the context of GGNet. Recently, these works have been utilized by the wheat community. For example, Liu et al. combined AHG and gHap to identify selection signals during the evolutionary process and evaluate the utility of the *TaTPP-7A*-belonging genome region in breeding (Liu et al. 2023).

The pervasive large CNV blocks in wheat varieties were a specific characteristic as a result of the frequent intra- and inter-species introgressions and crossings with relative species during modern breeding, largely expanding the range of genetic diversity. To fully profile those CNV blocks among germplasms, we identified a representative panel of large CNV blocks as novel markers, some of which were able to be associated with known chromosome aberrations and functional haplotypes, such as the important introgressions 1BL/1RS and 2N^v^S. With these new in silico markers, researchers can accurately scan the presence/absence variations (PAVs) of markers with ultra-low coverage (supporting a minimum of 0.05 ×) WGS data, further profile potential introgression fragments, and determine the authenticity of the interested materials. A CNV block-based marker atlas covering more than one thousand wheat varieties was constructed, together with an online database, WheatCNVb, to help the public freely access the CNV block profiles within the resequencing panel and perform customized analyses on uncatalogued materials.

### Connect emerging assemblies by genes

Increasing genome assemblies in Triticeae provide great opportunities for wheat research, such as uncovering the role of functional genes in shaping phenotypic variation and driving plant evolution. To fully explore the available genomic resources, we have developed a tool, GeneTribe, for homology inference among multiple assemblies in the pan-genomic era (Chen et al. 2020) (Fig. 2C). Considering that the Triticeae tribe is a typical allopolyploid-rich clade with reticulated evolutionary relationships (Feldman and Levy 2015), a polyploid assembly can be decomposed into multiple diploid subassemblies. GeneTribe can dynamically integrate collinear blocks and sequence similarity to enhance the power of homology inference. Additionally, a computationally unambiguous data structure was introduced to avoid overinterpretation of the homology data. Finally, the homology and collinearity data across Triticeae genomes generated by GeneTribe was built into an online database, Triticeae-GeneTribe (TGT) (Fig. 2C). This database provides users with homology and collinearity analysis of published genomes, assisting users in elucidating the origins and evolutionary histories of functional genes. The design scheme of TGT is suitable for rapid integration of published genomes. Since its release, the number of genomes in this database has increased to more than 80, encompassing all published high-quality assemblies in Triticeae. This work serves as a practical guide for other species to explore pan-genomic resources. Recently, this workflow has been applied to the genomic analysis of rice, promoting the construction of the Rice Gene Index database to provide richer analysis capabilities for the whole rice research community (Yu et al. 2023).

Some gene families with peculiar structures and features contribute to wheat evolution, necessitating novel methodological strategies to investigate them. A representative example is the intracellular gene transfer (IGT) from organelles to the nucleus, also known as endosymbiotic gene transfer, which is an ongoing process in plants (Kleine et al. 2009; Sloan et al. 2018). However, the widespread presence of these genes in the genome, coupled with poor annotation quality and high sequence similarity among IGTs, makes evolutionary analyses particularly difficult. We have developed a tool, IGTminer, that employs gene reannotation and collinearity information to identify the homologous and collinear IGTs and reveal the evolutionary trajectories of IGTs at the pangenomic level (Chen et al. 2023c) (Fig. 2C). We then utilized IGTminer to construct an online database, pNOGmap, that contains the identification and evolutionary trajectories of NOGs among the 67 genomes of 15 Poaceae species, including wheat and its relatives. The tool provides rich analytic and visualization functions to explore IGTs for better understanding genome variation and evolution in wheat and its relatives.

### Discover gene functions and regulations through networks

Discovering functional genes and regulatory mechanisms that control phenotypic variation can accelerate wheat improvement (Adamski et al. 2020; Wang et al. 2023), but the function of most genes in wheat remains unknown. Enormous amounts of omics data have not been effectively explored or utilized yet. We constructed a wheat integrative gene regulatory network (wGRN) by combining various functional information to provide a whole view of genes and their pathways controlling important traits (Chen et al. 2023b) (Fig. 2D). Considering that genome annotation greatly influences expression quantification and network construction, we presented an updated annotation by correcting gene models and adding new ones. Additionally, we utilized gene expression networks and physical regulatory networks, which complement each other when performing integrative regulatory network inference. Evolutionarily conserved regulation, which is under higher selective pressure and plays an important role in plants, was used as supporting evidence for network construction. Finally, a weighted integration approach was used to combine different supporting evidence and construct wGRN. An online platform was developed for users to utilize wGRN in terms of discovering gene function and regulation (Fig. 2D). The tool provides an effective method for identifying wheat gene functions using systems biology approaches, thereby greatly accelerating the efficiency of subsequent experiments and wheat improvement. wGRN can also provide a prioritization of candidate genes for gene mapping studies such as GWAS, expanding the list of candidate trait-associated genes. In addition, wGRN provides a powerful solution for analyzing emerging independent datasets in each lab. Overall, this platform indicates directions for the utilization of big data to guide functional gene discovery and crop improvement.

### Multi-omics tools and databases

Collaborative efforts in data interpretation and sharing are crucial when wheat omics data is accumulating rapidly. Genome browsers, such as JBrowse (Diesh et al. 2023), can offer graphical representations of omics data and make them more accessible and interpretable, allowing users to interactively conduct gene annotation, function analysis, and variation analysis. Due to the high cost of reanalyzing the omics data of polyploid wheat, integrating omics data generated from individual labs into a genome browser is an efficient way to promote scientific data sharing and collaboration. Our recent study performed ribosome profiling and polysome profiling to obtain a unique, comprehensive translatomic data set of developing bread wheat grains (Guo et al. 2023). The wheat grain translatome browser was implemented for the community to conveniently access the resources containing gene expression data at the translational level and the newly annotated ORFs, such as long non-coding RNAs (lncRNAs) and upstream open reading frames (uORFs). wLNCdb, a comprehensive database of wheat lncRNAs, was developed for users to investigate the function of lncRNAs (Zhang et al. 2023). Additionally, the WheatOmics database was developed to effectively integrate multi-omics data and several practical toolkits for the community to explore gene function information (Ma et al. 2021). These tools provide valuable resources for genomics-based design breeding.

## Guides to utilizing recent genomic tools and resources

### The dissection of the genetic basis underlying elite varieties

**Fig. 3:**
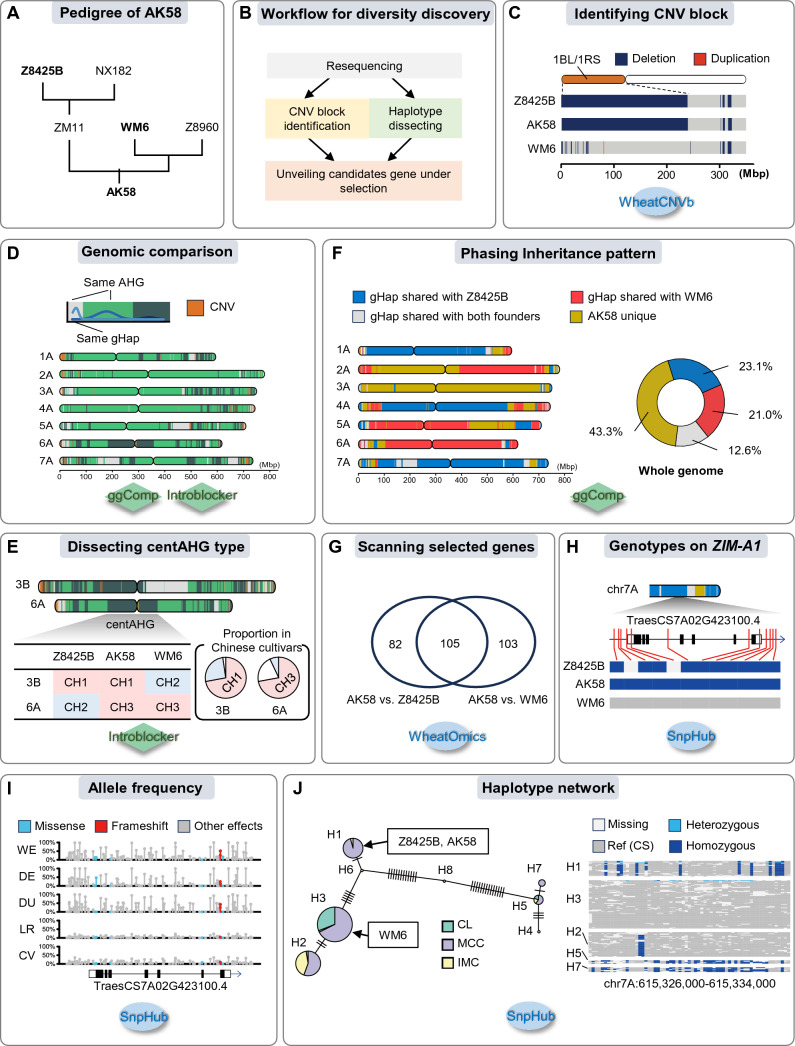
Genomic tools accelerate the discovery of genetic diversity between materials. **A** The pedigree of Bainong Aikang 58. Z8425B, Zhou 8425B. NX182, Neixiang 182. ZM11, Zhoumai 11. WM6, Wenmai 6. Z8960, Zheng 8960. AK58, Bainong Aikang 58. **B** Workflow for determining genomic diversity among AK58 and two of its founders, namely Z8425B and WM6. wheat-SnpHub-portal, WheatCompDB, and WheatCNVb were utilized to access and analyze wheat variation data, dissect haplotypes, and identify CNV blocks, respectively. **C** The CNV block patterns of Z8425B, AK58, and WM6 on the genome region corresponding to 1BS. Blue, deletion. Red, duplication. **D** Genomic comparison between Z8425B and WM6 in sliding windows along chromosomes. Gray, same gHap (the genetic resource between two accessions was accumulated just during breeding progress). Light green, same AHG (the diversity accumulated during domestication). Dark green, different AHG (the genetic types carried by Z8425B and WM6 diverged before domestication). Orange, different CNV block type. **E** CentAHG types on the chromosomes 3B and 6A. The pie chart shows the proportion of each centAHG type in Chinese cultivars. **F** Tracing the genome inheritance patterns of AK58 using gHap. Bule, genomic intervals with the same gHap between Z8425B and AK58. Red, intervals with the same gHap between WM6 and AK58. Gray, intervals with the same gHap among Z8425B, WM6, and AK58. Dark yellow, intervals with an AK58-specific gHap. **G** The Venn diagram shows the overlap of the characterized genes with different haplotypes between AK58 and Z8425B and that between AK58 and WM6. Genes were downloaded from the Known Gene page of WheatOmics. **H** Variation and haplotypes in the *ZIM-A1* region. Gray, genotype identical to Chinese Spring (CS). Dark blue, homozygous variation. Light gray, missing data. **I** The comparison of variation frequency between different wheat groups. Variation data containing tetraploid and hexaploid wheat was acquired from the wheat-SnpHub-portal. WE, wild emmer wheat. DE, domesticated emmer wheat. DU, durum wheat. LR, landrace of hexaploid wheat. CV, cultivar of hexaploid wheat. Light blue, missense variant that would cause a codon that produces a different amino. Red, frameshift variant that would cause a frame shift. Gray, variants causing no effects on coding. **J** Haplotype network and genotype heatmap of *ZIM-A1* in Chinese wheat collection. Green, Chinese landrace (CL). Purple, modern Chinese cultivar (MCC). Yellow, introduced modern cultivar (IMC). Dark gray, genotype identical to CS. Light blue, heterozygous variation. Dark blue, homozygous variation

Recombination is the basis of breeding programs to integrate desired traits together, and identifying haplotypes used by breeders is therefore valuable for future breeding (Bevan et al. 2017). With our recently developed genomics tools, it is now possible to examine the diversity between accessions at resolutions as fine as both Mb and single nucleotides. Here, we demonstrate the application of these tools to dissecting the genetic foundations of elite varieties (Fig. 3). Bainong Aikang 58 (AK58), a widely planted cultivar and an important founder in Chinese modern breeding, along with two of its founders, the well-known and important varieties Zhou 8425B (Z8425B) and Wenmai 6 (WM6, also known as Yumai 49), were taken as an example (Fig. 3A and B).

To determine the genomic regions with diversity, we first calculated the CNV-based similarity among these three accessions, resulting in a higher similarity between AK58 and Z8425B (~ 44.6%) compared to AK58 and WM6 (~ 28.8%), indicating that AK58 might have inherited more structure variations from Z8425B. A PAV on the well-known 1BL/1RS translocation was also identified by WheatCNVb (Fig. 3C). Considering the significant contribution of 1BL/1RS translocation to grain yield and biotic resistance (Wang et al. 2017), Z8425B and AK58 would have a competitive edge over other varieties. Haplotype diversity between Z8425B and WM6 was then analyzed using ggComp and IntroBlocker (Fig. 3D), resulting in that only 16.9% of the genome sharing the same gHap (corresponding to diversification that occurred during the breeding process, colored in gray) in two varieties and 16.1% of the genome being classified into different AHGs (indicating a difference in wild emmer ancient, colored in dark green). On chromosomes 3B and 6A, two large intervals overlapping with the centromeric region carrying different AHG types can be observed, suggesting a diversity in centAHG type between the two founders (Fig. 3E). According to our previous study (Wang et al. 2022), the common centAHG types of 3B and 6A in Chinese cultivars are centAHG type 1 (CH1) and CH3, respectively. The success of AK58 as an important founder can partly be attributed to the integration of the common centAHG type, considering that centAHG may act as the chromosome backbone that influences synapsis (Fig. 3E). To further elucidate the genetic contribution of the two founders, we identified the inheritance patterns on the AK58 genome with gHap consistency (Fig. 3F) and observed that the contributions of Z8425B and WM6 were almost identical, while 43.3% of the AK58 genome was inherited from the other two founders, suggesting a broad genetic source for AK58.

To locate candidate genes that were selected during the foundation of AK58, we scanned the genotype of cloned genes cataloged by WheatOmics (Ma et al. 2021). Our analysis showed that out of the 487 known genes, 187 exhibited variations between Z8425B and AK58, while 208 had variations between WM6 and AK58 (Fig. 3G) involving *TaZIM-A1*, a gene influencing heading date and grain weight (Liu et al. 2019) (Fig. 3H). The variation frequency analysis of *TaZIM-A1* (Fig. 3I) shows that the average frequency of non-CS alleles is relatively higher in tetraploid wheats (wild emmer, domesticated emmer, and durum wheat) but dramatically decreased in hexaploid wheats (landrace and cultivar), indicating the existence of selection pressure or the founder effect. To find out the favored haplotype in bread wheat, we used the HapNet module and the Heatmap module in SnpHub to investigate the evolutionary relationships among the *ZIM-A1* haplotypes in Chinese germplasm (Fig. 3J). WM6 can be assigned to haplotype 3 (H3), which represents the same type in Chinese Spring (CS), and Z8425B and AK58 were assigned to haplotype H1 (Fig. 3J). The haplotype network also reflected that most Chinese landraces (CL) carried H3, while modern Chinese cultivars (MCC) had a higher proportion of H1, H2, H5, and H7, suggesting that these haplotypes were selected during modern breeding progress in China, which is consistent with previous studies (Liu et al. 2019). Therefore, the H1 carried by AK58 may contribute to a higher breeding value. Overall, this case can serve as a route map for dissecting the genetic basis of elite cultivars.

### Functional gene discovery and characterization

**Fig. 4:**
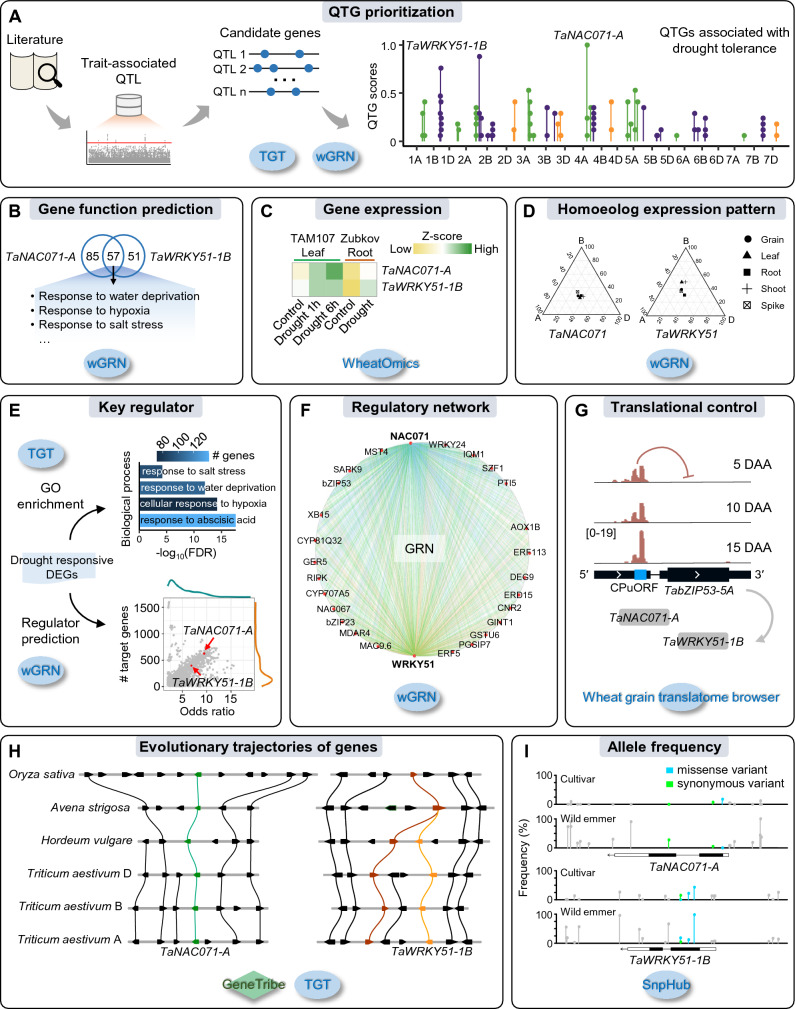
Genomic tools accelerate the discovery and characterization of functional genes. **A** Quantitative trait gene (QTG) prioritization for wheat drought tolerance GWAS. The QTGminer module in wGRN was used to prioritize candidates in QTL, and the GeneCard module in TGT can be utilized to acquire gene annotation. The QTL were acquired from two previous studies (Li et al. 2019; Mao et al. 2022). Higher QTG scores represent higher trait associations. **B** The Venn diagram shows the overlap of the genes’ predicted biological processes by the “function inference” module in wGRN. **C** The heat map shows expression levels of the genes in the drought-treated wheat plants, acquired from the “Gene Expression” module in WheatOmics. **D** The ternary plot shows relative expression levels of the triad in different tissues, conducted by the “homoeolog triad” module in wGRN. **E** The GO enrichment analysis was conducted by the “GO enrichment” module in TGT, and the regulator prediction analysis was conducted by the “TF enrichment” module in wGRN. The differentially expressed genes under drought stress are as inputs into tools. **F** The regulatory relationships between transcription factors and targets predicted by the “Regulation search” in wGRN. Selected genes were highlighted. **G** The expression of a uORF and its effect on the translation of the *TabZIP53-5A* mORF, acquired from the wheat grain translatome browser. *TabZIP53-5A* was predicted to be an upstream regulator of two candidate genes. DAA represents day after anthesis. **H** The microcollinearity visualization shows genes on the local scale among the three wheat subgenomes, barley, oat, and rice genomes. The analysis was done using the Microcollinearity and Homologues tools in TGT. The two panels show the collinear blocks containing *TaNAC071-A* and *TaWRKY51-1B*, respectively. The one-to-one homologous relationships were shown. The direction of the tracks was reversed manually for visualization. **I** The allele frequency of genomic variants within the genomic region of two candidate genes, done by the SnpFreq module in the wheat SnpHub portal. The colors indicate the effect of variants

The understanding of agronomic trait biology has been constrained by the intricate genome composition and time-consuming experiments. Our recently developed tool and databases can assist users in discovering functional genes and their regulatory mechanisms. We illustrated an example of the identification and characterization of drought tolerance-associated genes to demonstrate how to apply these analysis tools (Fig. 4). We performed a literature search and collected 97 drought stress (DS)-associated QTL from two previous studies on wheat GWAS (Li et al. 2019; Mao et al. 2022). We employed the QTGminer module in wGRN to prioritize DS-associated candidates (Fig. 4A). Combined with the distribution of QTG scores, we used the GeneCard module in TGT to screen the annotation of the candidate genes with high scores, focusing on the two high-confidence candidates, namely *TaWRKY51-1B* (TraesCS1B02G374900) and *TaNAC071-A* (TraesCS4A02G219700). By further searching for homologs of these two genes using TGT, we found that *TaNAC071-A* is known to be involved in drought tolerance (Mao et al. 2022), and *WRKY51* in other species is known to be involved in abiotic stress (Phukan et al. 2016; Zhou et al. 2023), supporting the accuracy of the results from QTGminer.

To conduct an analysis of the biologic function of these two genes, we used the “Function inference” module in wGRN to search for the pathways that *TaWRKY51-1B* and *TaNAC071-A* may be involved in. The overlap of the predicted functions of *TaWRKY51-1B* and *TaNAC071-A* includes several terms associated with abiotic stress, such as response to water deprivation, response to hypoxia, and response to salt stress (Fig. 4B). By employing the “Gene Expression” module in WheatOmics, we found that both genes were differentially expressed under drought stress (Fig. 4C). The homoeolog expression bias patterns of the genes conducted by the “Homoeolog Expression” module in wGRN showed the similar expression and function conservation of the homoeologs (Fig. 4D).

We investigated the drought stress-associated regulatory networks involving these two genes. According to the “Gene Browser” module in wGRN, *TaWRKY51-1B* and *TaNAC071-A* were classified into the WRKY and NAC transcription factor families. To further reveal the position of genes in the DS regulatory network, we acquired an RNA-seq data set of drought-treated wheat (Liu et al. 2015) and identified differentially expressed genes (DEGs) using a previous pipeline (Chen et al. 2023b). Taking the DEGs as input, the “GO enrichment” module in TGT revealed the expected terms, such as response to water deprivation (Fig. 4E). The “Regulator prediction” module in wGRN showed that the two candidates were key regulators of wheat drought tolerance (Fig. 4E). We further used the Search module in wGRN to identify the two genes’ potential upstream regulators and downstream targets, some of which have been reported to be involved in plant abiotic stress, suggesting that *TaWRKY51-1B* and *TaNAC071-A* mediate drought tolerance-associated regulatory networks (Fig. 4F). Notably, the wheat grain translatome browser revealed that the translation of *TabZIP53-5A*, an upstream regulator of the two genes, was repressed by its uORF (Fig. 4G). The uORF is conserved in plants (Guo et al. 2023; Juntawong et al. 2014), and hypoxia alters the translation of the uORF relative to the *bZIP53* mORF (Juntawong et al. 2014). These findings suggest that *TaWRKY51-1B* and *TaNAC071-A* are involved in drought-responsive regulatory networks at both the transcriptional and translational levels.

We next analyzed the inter- and intraspecies genomic variation and evolution of the genes. We combined the Homologues and Micro-collinearity tools in TGT to identify orthologs and paralogs of the genes among four representative Poaceae crops, including wheat, barley, oat, and rice (Fig. 4H). The results show that *TaNAC071-A* is conserved among Poaceae species and has only one copy in each wheat subgenome. *TaWRKY51-1B* is specifically present in the tested Triticeae crops and is a paralog of its flanking gene; that is, the homologs are tandemly duplicated in the three wheat subgenomes and barley. *TaWRKY51-1B* and *TaNAC071-A* may have different evolutionary histories and origins. By accessing the SnpFreq module in SnpHub, we observed different allele frequencies of the genes between the wild emmer tetraploid wheat and hexaploid wheat cultivar groups, indicating that they may have undergone strong selection during the evolution of wheat germplasm resources (Fig. 4I). Overall, the analysis conducted using genomic tools and databases sheds new insights on wheat drought tolerance.

## Future perspectives

### Comprehensive and cost-efficient genomic variation detection

**Fig. 5:**
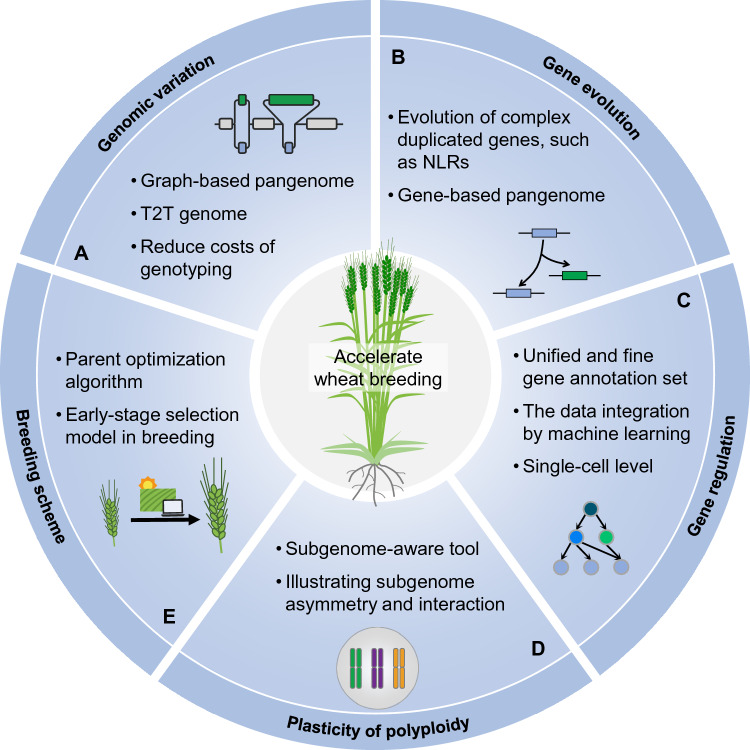
Future perspectives in tool development for wheat. **A** Genomic variation. Capturing a more complete picture of genomic variation within populations is essential for population genetics research and gene cloning for wheat improvement. New methods are needed for cutting costs in genotyping and for identifying fine genomic diversity. **B** Gene evolution. Deciphering the evolution of highly duplicated complex gene families and constructing a high-resolution gene-based pangenome requires considering the unique characteristics of each gene family, thereby enhancing our understanding of important genes such as NLRs. **C** Gene regulation. An in-depth understanding of the regulatory mechanisms of complex agronomic traits at a systems level requires not only a fine and unified annotation set but also comprehensive consideration of regulatory effects beyond transcriptional levels. Additionally, machine learning methods are needed to integrate data with high cellular and spatiotemporal resolutions. **D** Genomic plasticity of ployploid wheat. Polyploidizations that have occurred in wheat have contributed to important agronomic traits. Subgenome dominance and asymmetry in hexaploid bread wheat need further investigation. Subgenome-aware tools and techniques could assist in unveiling the factors contributing to the success of polyploid bread wheat. **E** Breeding scheme. Parental selection can greatly contribute to the efficiency of the breeding process. However, optimization strategies for modern breeding schemes that utilize big omics data and machine learning algorithms are still lacking in wheat

The cost of WGS is a significant obstacle, considering that even though the price for sequencing could be as cheap as 10 yuan RMB (~ $1.4) per Gb on the HiSeq X Ten platform, about 1000 yuan RMB (~ $140) is still needed for performing a 6 × WGS in wheat. While low-coverage WGS has been successfully applied to predict founder haplotypes on the genome of offspring from a wheat Multiparent Advanced Generation Intercross (MAGIC) population (Scott et al. 2021), such a strategy has not yet been applied to a population with a more complex genetic background, such as a natural population. Additionally, sequencing reads from the genomic region of one subgenome may be falsely aligned to another subgenome in polyploid wheat, although such regions only make up a small portion due to genetic diversity among the diploid lineages. Efforts to improve the accuracy of variant calling could always help understand functional genomes more clearly, such as through long-read sequencing. A more cost-efficient and accurate strategy for genotyping is needed by future innovations in experimental and analytical technologies (Fig. 5A).

Challenges also exist in mining and tracing favorable alleles on agronomically important genes in the incompletely assembled regions. The nature of frequent introgressions in wheat poses limitations on comprehensively characterizing the genomic regions and genes of other species. The construction of the telomere-to-telomere (T2T) genome assembly could be one solution (Fig. 5A), which has already been achieved on crops such as soybean (Wang et al. 2023a), rice (Shang et al. 2023), and maize (Chen et al. 2023a). However, the final solution should be building a comprehensive and complete wheat pan-genome, or even a Triticeae pan-genome (Fig. 5A). To achieve such a goal, the assembly accuracy in complex genome regions with numerous mutations and duplications still needs improvement (Liao et al. 2023). In summary, more robust and cost-effective sequencing and analysis methods, as well as better assemblies, are still required to comprehensively characterize variations in the complex genome of wheat.

### Clarifying the complex gene evolution histories in polyploid wheat

Agronomically important gene families with a complex evolutionary history require greater understanding (Fig. 5B). New genes can be gained via whole genome duplications, tandem duplications, segmental duplications, TE-mediated duplications, introgression, gene transfer, and de novo gene birth. Gene duplication has been shown to play an important role in plant growth and environmental adaptation (Kuzmin et al. 2022; Ren et al. 2018; Wu et al. 2020). It is important to decipher the evolution of genes abundant in the genome, such as phylogeny and function divergence. However, many gene families that have specific sequence features and complex evolution histories have not been thoroughly analyzed in polyploid wheat. A typical example is the nucleotide-binding and leucine-rich repeat (NLR) genes, which are abundantly present and frequently duplicated, and half of these loci are at a distance of less than 50 kb from another locus (Steuernagel et al. 2020). The NLRs are typically tandemly duplicated in Triticeae, and the number gradually increased during the evolution and domestication of bread wheat (Chen et al. 2020). Advanced tools should be developed to finely analyze the evolutionary history of NLR genes in wheat. A representative pangenome resource allows a more complete picture of the evolutionary trajectories of genes and can provide new insights into how selection may lead to gene variation and evolution (Bayer et al. 2020). Additionally, the construction of high-quality T2T assemblies in Triticeae can help dissect extensive gene duplication and loss, thereby assisting in accurately characterizing gene duplications such as NLRs. Apart from assembly quality, low gene annotation quality has posed limitations on comparative genomic analysis, requiring new gene annotation pipelines to address this challenge. Integrating big data for a unified re-annotation of published genomes is an urgent goal. In addition to the graph-based pangenome, the construction of a gene-based pangenome that characterizes gene variation at either the population level or the species level is imperative (Fig. 5B). The systematic dissection of gene evolution histories is critical to the understanding of phenotypic evolution in polyploid wheat.

### High-resolution regulatory network architectures

Plants are organisms that involve the collaboration of many functional elements. Achieving a systematic-level understanding of gene function and regulation requires a high-quality genome annotation (Fig. 5C), which includes not only protein-coding genes but also non-coding RNA regions. The inadequate gene annotation has reduced the analysis power for gene function, variation, and evolution in polyploid wheat. Iso-Seq and Ribo-seq (ribosome profiling) can improve the annotation of transcribed and translated regions (Chen et al. 2023b; Guo et al. 2023). In the future, it is essential to develop a unified and customized gene annotation pipeline for wheat and its relatives, thereby achieving a comprehensive annotation of wheat gene structures. Notably, the ideal construction of a regulatory network should involve multiple regulatory layers and consider the temporal and spatial expression patterns, as well as variations between genotypes and those between subgenomes, of all functional elements (Fig. 5C). Previous research often focused solely on the transcriptional level, overlooking other levels such as post-transcriptional, translational, and post-translational regulations (Ingolia 2016; Lavarenne et al. 2018; Mergner et al. 2020). The utilization of tissue- and stage-specific data as well as single-cell and spatial transcriptomic data, considering the heterogeneity between cells, can help exploit regulatory processes in complex traits. In addition, using genomic variation and phenotypic data to unveil the impact of variation on gene regulation can aid in dissecting the genotype-specific regulatory networks contributing to phenotypic variation. A high-quality T2T genome and even a more comprehensive pan-genome can help better decipher regulatory network variations between different wheat accessions. However, a significant question is whether we can extract biologically meaningful insights from highly dimensional datasets at a reasonable computational cost (Ko and Brandizzi 2020). Machine learning can provide powerful solutions for the comprehensive analysis of large and heterogeneous datasets (Wong et al. 2021; Wu et al. 2021). The training datasets (known information) required for machine learning are scarce, thereby limiting its application in wheat. With the utilization of CRISPR gene editing and genomics technologies, the number of known genes is expected to increase in the future (Gao 2021). Constructing a high-resolution regulatory network can help get a deeper understanding of agronomic trait biology in polyploid wheat.

### Charting subgenome interaction and asymmetry after polyploidization

Polyploidization, or WGD, has been proposed as the major force driving the evolution of flowering plants. Gene retention, functional divergence, and regulatory network complexity after polyploidization result in key phenotypic innovations (Jiao 2018; Soltis and Soltis 2016). Allohexaploid bread wheat must coordinate subgenomes with distinct genetic and epigenetic landscapes within a single nucleus (Fig. 5D). The presence of homoeologs in polyploid wheat confers adaptive plasticity through expression divergence and neofunctionalization (Pfeifer et al. 2014; Ramirez-Gonzalez et al. 2018). The gene redundancy that arises from WGD events can buffer the deleterious effect of genome variations (Uauy 2017). Benefiting from high-throughput sequencing technology, subgenome dominance and asymmetry of gene expression, regulation, and translation in hexaploid bread wheat are now beginning to be revealed (Guo et al. 2023; Ramirez-Gonzalez et al. 2018). The application of multi-omics big data will further deepen the understanding of subgenome interaction. In the future, the development of subgenome-aware tools and techniques is key to accurately identifying subgenome-specific variations and functions, thereby better understanding subgenome asymmetry and evolution after polyploidization and further unveiling the factors contributing to the success of polyploid bread wheat. Understanding the coordinated expression and regulation of homoeologs between the subgenomes in polyploid wheat could help define strategies to improve agronomic traits.

### Accurate wheat breeding scheme optimization

Genomics-assisted breeding (GAB) has been demonstrated to play a crucial role in the development of elite cultivars (Varshney et al. 2021). However, compared with the other two major crops, rice and maize, the algorithms and strategies for GAB are largely lacking in wheat. In rice, the RiceNavi system was developed to optimize breeding routes using a comprehensive catalog of causative variants (Wei et al. 2021). A selection strategy in maize, target-oriented prioritization, was designed to identify candidate hybrids meeting a phenotypical breeding goal (Yang et al. 2022a). However, such methodology is lacking in wheat due to the limited number of cloned genes and the functional redundancy caused by the polyploidy nature. To accelerate breeding in wheat, it is necessary to extract valuable information from published research on wheat to develop a knowledge database for hosting and integrating gene functions, QTL, phenotypes, and other relevant information. In the future, new methods and strategies, such as advanced machine learning algorithms, evolutionary algorithm-powered genome selection strategies, and fully representative databases of germplasm resources, are required for improving efficiency and reducing costs in the functional gene mapping process, thereby improving breeding schemes to acerate wheat breeding into the 4.0 era (Fig. 5E).

## Conclusions

Massive biological data enables the community to get a better understanding of the function, regulation, variation, and evolution of genes involved in important traits, such as resistance, adaptability, and yield. However, the huge and complex genome of bread wheat poses constraints on its functional genomics and genetic improvement. How to mine and utilize such data is a pressing issue faced by researchers. Recently, we have developed a series of innovative computational tools and databases to provide excellent demonstrations for deciphering the bread wheat genome and distilling biologically meaningful knowledge from big data, thereby assisting in functional genome dissection and variety improvement. To demonstrate the potential of the tools, we presented two cases for analysis on dissecting the genetic diversity within a pedigree, and unveiling the functional genes associated with drought tolerance, respectively. These analytical cases were conducted by utilizing multiple analysis tools and databases, without the need for experiments, indicating that these tools can provide convenience for breeders and molecular biologists to dissect the functional genome of wheat. We also discuss potential routes for designing future genomic tools that specifically target the unique characteristics of the wheat genome. Developing dedicated tools and databases is an effective solution to perform in-depth analysis and integration of big biology data. The combination of experiments and bioinformatics knowledge can accelerate the transformation of function information into breeding efforts. The utilization of efficient and high-performance tools and databases will drive a transformation in wheat breeding technologies, thereby enhancing the development of superior wheat varieties to meet the increasing food demand of the growing population under ever-changing climatic conditions.

## Data Availability

The pipelines and commands for the step-by-step examples are available through the website link: http://wheat.cau.edu.cn/resources/tutorial.html. The mentioned tools and databases are summarized in Table 1.
